# Quantitative determination of major alkaloids in *Cinchona* bark by Supercritical Fluid Chromatography

**DOI:** 10.1016/j.chroma.2018.04.038

**Published:** 2018-04-18

**Authors:** Adele Murauer, Markus Ganzera

**Affiliations:** Institute of Pharmacy, Pharmacognosy, Center for Molecular Biosciences (CMBI), University of Innsbruck, Innsbruck, Austria

**Keywords:** *Cinchona* sp., *Cinchona* bark, Supercritical Fluid Chromatography, Chinoline alkaloids, Quinine, Quantification

## Abstract

Chinoline alkaloids found in *Cinchona* bark still play an important role in medicine, for example as anti-malarial and antiarrhythmic drugs. For the first time Supercritical Fluid Chromatography has been utilized for their separation. Six respective derivatives (dihydroquinidine, dihydroquinine, quinidine, quinine, cinchonine and cinchonidine) could be resolved in less than 7 min, and three of them quantified in crude plant extracts. The optimum stationary phase showed to be an Acquity UPC^2^ Torus DEA 1.7 µm column, the mobile phase comprised of CO_2_, acetonitrile, methanol and diethylamine. Method validation confirmed that the procedure is selective, accurate (recovery rates from 97.2% to 103.7%), precise (intra-day ≤2.2%, inter-day ≤3.0%) and linear (R^2^ ≥ 0.999); at 275 nm the observed detection limits were always below 2.5 µg/ml. In all of the samples analyzed cinchonine dominated (1.87%–2.30%), followed by quinine and cinchonidine. Their total content ranged from 4.75% to 5.20%. These values are in good agreement with published data, so that due to unmatched speed and environmental friendly character SFC is definitely an excellent alternative for the analysis of these important natural products.

## 1 Introduction

Cinchonae cortex, which originates from several related species of the genus *Cinchona* (*C. pubescens*, *C. calisaya*, *C. ledgeriana* and hybrids) according to the European Pharmacopeia, was used as antimalarial drug by the indigenous population of South America for centuries. It became the primary remedy against this disease worldwide, and only after World War 2 synthetic antimalarials like chloroquine replaced the natural product [1]. However, due to increasing resistances and also availability issues quinine is still relevant for malaria treatment today [2]; besides that the compound is added to beverages as bitter agent [3], serves a catalyst in asymmetric organic synthesis [4], or acts as chiral selector in stationary phases [5]. The alkaloid pattern in *Cinchona* bark is rather complex with more than 30 known representatives [6]. They mainly are chinoline derivatives, including the diastereomeric pairs quinine/quinidine and cinchonine/cinchonidine. Additional alkaloids are, among others, their dihydro-derivatives.

Not only due to the medicinal and commercial importance of *Cinchona* bark but also the narrow therapeutic window of quinine many analytical studies focused on the determination of alkaloids in the crude drug. The compendial method in the 9th edition of the Ph.Eu. is based on a photometric determination of quinine (348 nm) and cinchonine-type (316 nm) alkaloids. Research papers mainly emphasized on the separation of the dominant representatives utilizing TLC [7], isotachophoresis [8], aqueous [9] and non-aqueous CE [10], vibrational spectroscopy [11], NMR [12] and HPLC [3,6,13–16]. For example, Hoffmann et al. utilized a chiral strong cation exchange material to excellently resolve eight *Cinchona* alkaloids in 15 min, yet an application to plant material is missing [16]. The latter was presented in the most recent study, in which Holmfred et al. reported on the separation of the four main isomers on 2.6 µm C-18 core shell material (Kinetex XB-C18) in 25 min [17]. Whether Supercritical Fluid Chromatography (SFC) is a possible and equivalent alternative has never been investigated. In the past the technique was predominantly used for the analysis of non-polar compounds like fatty acids [18], triglycerides [19] or carotenoids [20]. Recent publications point to a much wider range of possible applications also including polar natural products [21,22] and alkaloids [23–25]. Therefore, we attempted to separate and quantify the alkaloids in *Cinchona* bark by SFC.

## 2 Materials and methods

### 2.1 Standards and reagents

**Fig. 1:**
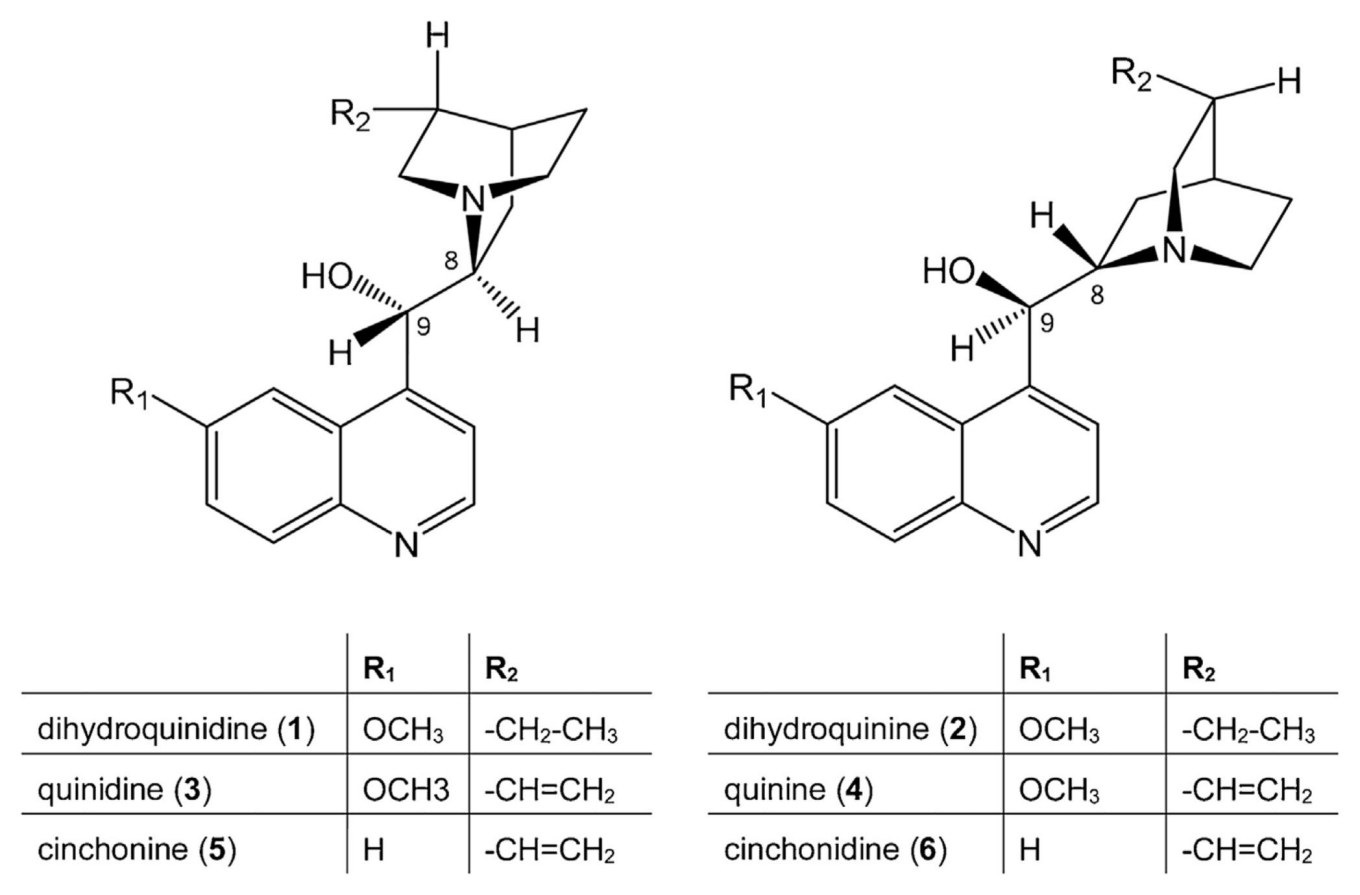
Chemical structure of the assayed *Cinchona* alkaloids.

Six *Cinchona* alkaloids (compounds **1**-**6**, see Fig. 1 for structures) with a purity ≥98% were available as standards; they were purchased from Phytolab (Vestenbergsreuth, Germany; compounds **1** and **2**) and Sigma Aldrich (St. Louis, MO, USA; compounds **3**-**6**). Plant samples (CC-2017-1 to CC-2017-4) were bought 2017 in different pharmacies in Innsbruck, Austria; voucher specimens are deposited at the Institute of Pharmacy, Pharmacognosy, University of Innsbruck. Compressed carbon dioxide for SFC analysis had a purity of ≥99.995% (4.5 grade) and came from Messer (Gumpoldskirchen, Austria). All solvents and reagents (methanol, acetonitrile, diethylamine, trimethylamine, sodium hydroxide, acetic acid, ammonium acetate, phosphoric acid) utilized in this study were of analytical grade and purchased from Merck (Darmstadt, Germany). An Arium 611 water purification system from Sartorius (Göttingen, Germany) produced the required HPLC grade water.

### 2.2 Sample preparation

The plant material (Cinchonae cortex Ph.Eu.) was finely pulverized in a mill and 150 mg were extracted following a published protocol [6]. Extraction solvent was a methanol/0.1 M NaOH mixture in the ratio 49/1; the samples were extracted three-times with 10 ml of this mixture by sonication (Bandelin Sonorex, Berlin, Germany) for 20 min each. After each step they were centrifuged for 10 min at 1500*g*, and the clear supernatant combined in a 50 ml volumetric flask. Then the latter was filled to volume with the extraction solvent. Sample solutions were membrane filtered right before analysis (0.45 µm cellulose acetate membrane, VWR, Vienna, Austria) and injected in triplicate. If stored at 4 °C sample and standard solutions are stable for at least 2 weeks.

### 2.3 Analytical method

For all experiments an Acquity UPC^2^-SFC instrument from Waters (Milford, MA, USA), equipped with convergence manager, column oven, sample manager, binary solvent manager and PDA detector was used; the operating software was Empower 3. Optimum separation of the six standards was achieved on an Acquity UPC^2^ Torus DEA column (3.0 × 100 mm, 1.7 µm) from Waters, protected by a guard filter (critical clean; Waters). The mobile phase comprised CO_2_ (A) and 0.8% diethylamine in a mixture of 10% acetonitrile and 90% methanol (B). Isocratic separation was achieved by maintaining a concentration of 97.7A/2.3B over 10 min. The injected sample volume was 1 µl, while flow rate, column temperature and ABPR pressure were set to 1.8 ml/min, 15 °C and 150 bar (2175 psi). The compounds of interest were detected at 275 nm. The sample manager was maintained at 10 °C, and a mixture of methanol/2-propanol (1:1) and methanol served as a weak and strong wash, respectively.

### 2.4 Method validation

To assure that the developed SFC method conforms to regulatory standards it was validated according to ICH guidelines [26]. For the construction of calibration curves as well as to determine the linear range approximately 1 mg of each standard was accurately weighted and dissolved in 1 ml methanol (stock solution). This solution was used to prepare further calibration levels by serial dilution in the ratio of 1:1 with the same solvent. LOD (limit of detection) and LOQ (limit of quantification) values were calculated as described in the guidelines based on standard deviation of the response and slope of the calibration curve. Selectivity was confirmed by utilizing PDA data and the peak purity option of the operating software. Precision was assured by preparing and analyzing five solutions of sample CC-2017-2 on each of three consecutive days. Variations within one day (intra-day precision) and within three days (inter-day precision) were calculated based on the peak area. Accuracy was investigated by spiking sample CC-2017-2 with different concentrations of all standards (high, medium and low spike). Spiked samples were then extracted and analyzed as proposed. Recovery rates were calculated by comparing the actually found concentrations with the theoretically present ones. All results of the validation experiments are summarized in Table 1.

## 3 Results and discussion

Since its beginnings in the 1960s SFC has evolved into a widely utilized and efficient separation technique. A better understanding of the underlying theory, together with significantly improved instruments and stationary phases have led to many successful separations and a broad field of applications. However, relevant medicinal plants, whose ingredients seem to be not suitable for SFC because of their polarity, have never been investigated till date. One of them is *Cinchona* bark, a drug which is analytically challenging as it contains diastereomeric chinoline alkaloids as active constituents.

### 3.1 Method development

**Fig. 2:**
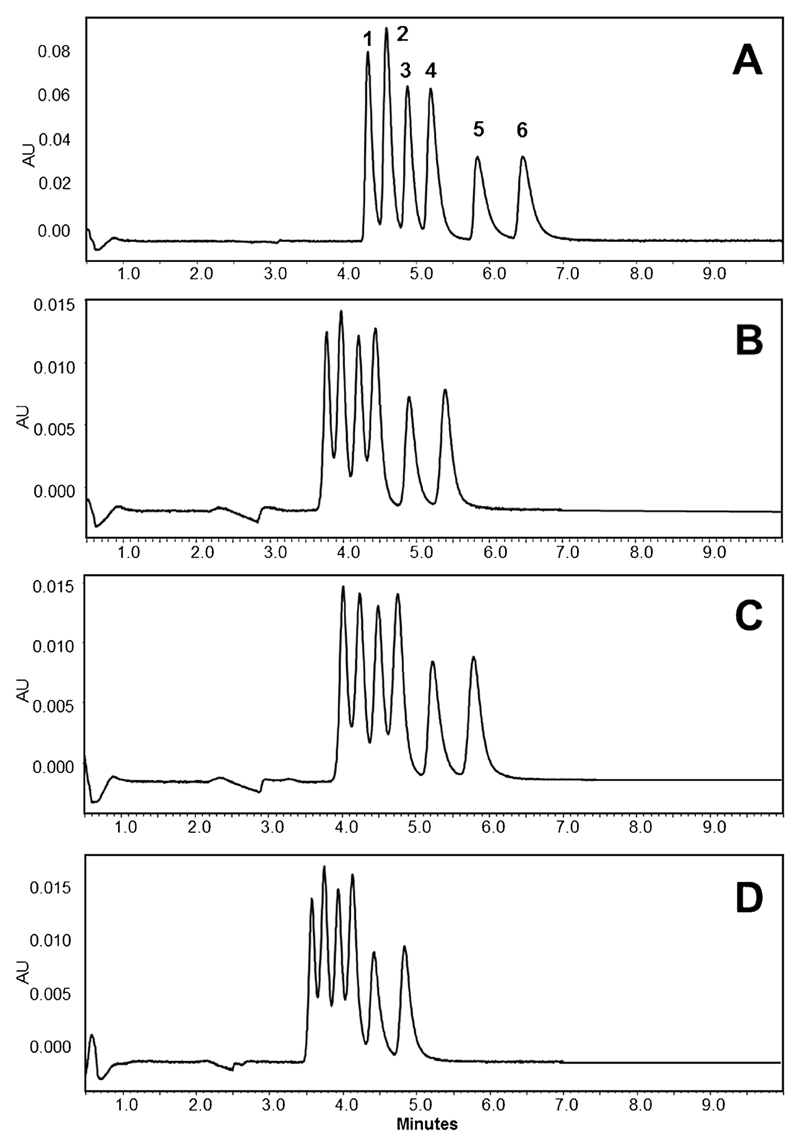
Separation of *Cinchona* alkaloids by SFC; optimum conditions (**A**; column: Acquity UPC^2^ Torus DEA 1.7 µm, 3.0 × 100 mm; mobile phase: CO_2_ (A) and 0.8% diethylamine in a mixture of 10% acetonitrile and 90% methanol (B); elution: isocratic with 97.7A/2.3B; sample volume: 1 µl; flow rate: 1.8 ml/min; column temperature: 15 °C; ABPR pressure: 150 bar; detection wavelength: 275 nm) and variations thereof: only MeOH and 0.8% DEA as modifier (**B**), isocratic elution with 2.5% B (**C**) and separation at 20 °C (**D**). Peak assignment is according to Fig. 1.

The optimum SFC separation of six major *Cinchona* alkaloids, namely dihydroquinidine (**1**), dihydroquinine (**2**), quinidine (**3**), quinine (**4**), cinchonine (**5**) and cinchonidine (**6**), within less than 7 min is shown in Fig. 2A. During method development it was observed that this result is only feasible by one specific combination of mobile and stationary phase. Concerning the latter, eight different SFC columns from Waters with identical dimensions (3.0 × 100 mm) and a particle size ≤2 µm were tested: four from the Torus series, i.e. 2-PIC, Diol, 1-AA and DEA, and four Viridis columns (BEH, BEH 2-EP, CSH Fluoro-Phenyl and HSS C18 SB). According to West and colleagues, who classified more than 30 ultra-high performance SFC stationary phases using a modified LSER (linear solvation energy relationship) model, from all the stationary phases available in this study Torus DEA (diethylamine) material has the highest basic character [27]. For this material the relevant a-term (basicity) is higher than 2.6, whereas for example for Viridis phases it ranges from 0.3 (CSH Fluoro-Phenyl) to 1.4 (BEH 2-EP). Accordingly, this material is designed to provide superior peak shape for bases [28]. With pKa values around 8.5 [29] the target analytes are such compounds, and therefore it seemed logic that this stationary phase was selected for further experiments. Only on Torus DEA material the compounds could be separated with acceptable resolution and peak shape, on others including all Viridis columns the compounds eluted as broad and overlapping signals only (see supporting information).

Concerning the mobile phase it was required to add organic solvents and diethylamine as modifiers. The polarity of pure CO_2_ is similar to hexane [30], and therefore a small percentage of methanol was required; the combination with acetonitrile was advantageous in terms of resolution (Fig. 2B), thus a MeOH/ACN mixture in the ratio of 9:1 was employed. However, without an alkaline eluent no acceptable result was possible. This observation was in agreement to literature, where an enhanced SFC separation of basic substances with an alkaline mobile phase is reported [31]. The authors attributed this effect to reduced secondary ionic interactions with residual silanols. For the current application the addition of 0.8% diethylamine (DEA) to the modifier (i.e. the aforementioned mixture of MeOH and ACN) showed to be the optimum. In terms of elution mode conditions had to be fine-tuned as well. Even with a very flat gradient the first four signals merged, so that isocratic conditions had to be selected; 97.7% phase A (CO_2_) and 2.3% B (MeOH, ACN and DEA) provided the best resolution. It is note-worthy to say that already a slight change (e.g. to 2.5% B; Fig. 2C) had a negative impact on the separation. Lowering the modifier concentration to 2.0% resulted in prolonged retention times, yet compounds **2** and **3** gradually overlapped.

Another parameter with significant influence on the separation of the six alkaloids was column temperature (Fig. 2D). Rather surprisingly, by lowering column temperature down to 15 °C retention times steadily increased. The opposite would be expected because at lower temperatures fluid density increases, resulting in reduced retention. A possible explanation for the observed effects might be changes in the polarity of the stationary phase due to a temperature-dependent adsorption of mobile phase components [32]. It is obvious that carbon dioxide was not present in the supercritical state anymore, because its critical temperature is 31 °C; however, working in the subcritical stage has no significant disadvantages and it is described (but not necessarily mentioned) quite often [33]. Further chromatograms indicating the relevance of individual method parameters are compiled as supplementary material. An interesting fact shown there is the influence of applied backpressure (ABPR). This setting is usually of minor importance, yet in the current application it modified resolution, particularly between compounds **2** and **3**. The latter could be resolved best at an applied ABPR of 150 bar.

### 3.2 Method validation

Assay development was followed by method validation; data presented in Table 1 confirms that all requirements were satisfactorily met in this respect. Selectivity was deduced by several facts. First, structurally closely related compounds (including diastereomers) could be resolved, second, no signs of co-elutions (e.g. peak shoulders) were visible, and third, the PDA data was very consistent within individual peaks. A final confirmation of peak purity by SFC-MS was not possible, because this technical option was not available. For all standards calibration curves were linear from approx. 1000–30 µg/ml, with determination coefficients always being higher than 0.999. LOD values showed to be in the range from 0.6 (**5**) to 2.4 (**2**) µg/ml, LOQ values varied from 1.9–7.3 µg/ml. They naturally cannot compete with those achievable by fluorescence detection (e.g. LOD for quinine is 2 fmol, [6]); however, they are comparable to conventional HPLC-UV as LOQ values of 5 µg/g are stated in reference [17]. Precision was investigated by repeatedly assaying sample CC-2017-2 under optimized extraction and separation conditions. Intra-day (≤2.2%) as well as inter-day variations (≤3.0%) were acceptable and typical for investigating plant material, which usually shows some degree of inhomogeneity. Last but not least, accuracy was determined in spiking experiments (high spike: 200 µg/ml, medium spike: 100 µg/ml, low spike: 50 µg/ml). Recovery rates were not lower than 97.2% (**3**, low spike) and not higher than 103.7% (**6**, high spike), indicating validity of this parameter too.

### 3.3 Analysis of the samples

Four samples of dried and milled *Cinchona* bark, all of them with Ph.Eu. quality, were available for quantification. Concerning the optimum extraction protocol a procedure described by Gatti et al. was adopted [6]. It utilizes alkaline methanol and sonication, and showed to be advantageous over others like soxhlet extraction in their work due to the mild conditions applied; the observed quantitative results were comparable. We modified the procedure in a way that sonication was repeated three times in order to assure exhaustiveness. The following facts support this estimation. First, if the remaining plant material is extracted and assayed one more time no remains of the alkaloids were detectable, and second, the excellent recovery rates already mentioned in the previous chapter.

**Fig. 3:**
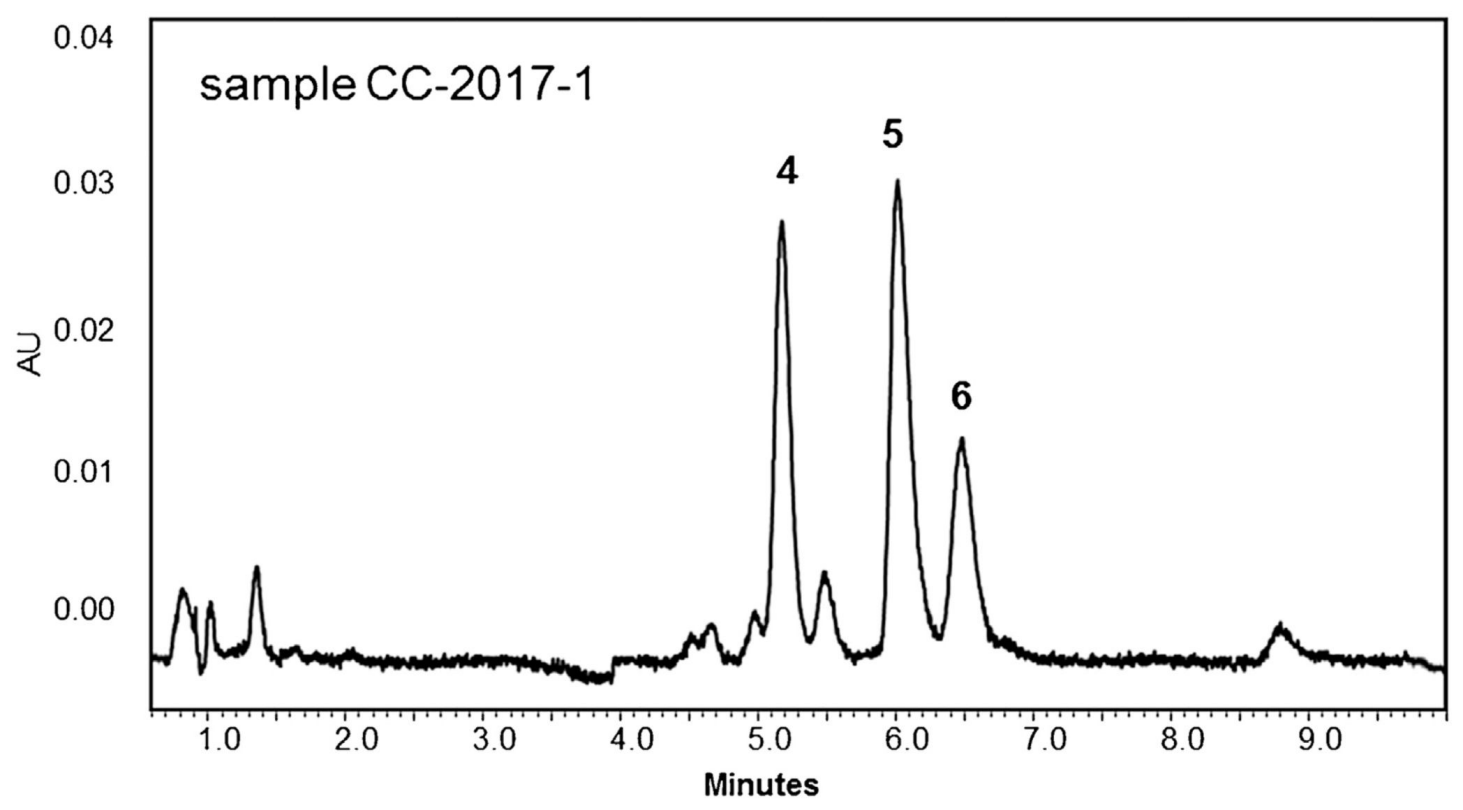
Analysis of sample CC-2017-1 under optimized SFC conditions (see Fig. 2). Peak assignment is according to Fig. 1.

A typical SFC chromatogram of a sample solution is shown in Fig. 3. The compiled quantitative results presented in Table 2 indicate that all of the investigated specimens were of similar composition. Three of the six standards were clearly assignable by matching retention times and UV-spectra; if these criteria were not met, e.g. peaks were too small for providing meaningful spectra, respective signals were not considered for quantitation. The assigned compounds were quinine, cinchonine and cinchonidine, with the latter always being the least abundant alkaloid (0.90%–1.26%). Most dominant was cinchonine (1.87%–2.30%), followed by quinine, which ranged from 1.59% to 1.89%; an excellent repeatability was observed while performing these experiments (σ_rel_ ≤ 1.55, n = 3). The total alkaloid content varied from 4.75% (sample CC-2017-3) to 5.20% (sample CC-2017-2).

## 4 Conclusion

This study is another proof for the excellent separation efficiency and versatility of SFC, especially in the field of natural products. The determination of alkaloids in *Cinchona* bark is a challenging task, because the target analytes are structurally very similar and the investigated matrix is complex like most biological samples. Due to the persisting practical relevance of the drug many attempts have been made to determine these compounds, mostly by using conventional RP-HPLC in combination with fluorescence detection. This assured an excellent sensitivity; however, the required analysis time was in the range from 15 [16] to 50 min [6], when only only recent publications are considered. That a comparable separation is also feasible in less than 7 min by using a “green technology” has been shown in the current study. This was only possible after meticulous method optimization, but once completed, a reproducible, accurate and rugged system was available for routine use; method validation confirmed this estimation. In the samples analyzed three out of six standards could be assigned. This is less than in previous reports, but explainable by the different detection techniques used. However, if suitable instrumentation is present (e.g. fluorescence detectors for SFC are available) there will be probably no difference in the number of identified compounds. With the available instrumentation quinine, cinchonine and cinchonidine could easily be assigned in crude *Cinchona* bark extracts. The quantitative results were well comparable to published data, which for example report the following values for a drug with Ph.EU. quality: 1.80% quinine, 1.65% cinchonine, and 1.25% cinchonidine [17]. This successful application of SFC, on for the utilized technique “untypical” compounds, should raise further interest to fully explore the potential of this separation technique, which definitely is not limited to the “classics” like carotenoids, fatty acids or terpenes. This and other studies on natural products like anthraquinones [34], kavalactones [35] or furocoumarins [36] are good indicators actually.

## Supplementary Material

Supplementary data associated with this article can be found, in the online version, at https://doi.org/10.1016/j.chroma.2018.04.038.

Supplementary Information

## Figures and Tables

**Table 1:** Results of method validation.

Regr. equation	1 y = 297.1 x +848.7	2 y = 315.1 x +182.1	3 y = 267.6 x −189.4	4 y = 273.4 x −34.1	5 y = 239.1 x −679.9	6 y = 252.8 x −724.0
R^2^	0.9998	0.9997	0.9996	0.9992	0.9994	0.9992
Linear range^a^	990–30.9	990–30.9	990–30.9	1020−31.9	1020−31.9	1010−31.6
LOD^a^	2.3	2.4	1.5	1.4	0.6	0.9
LOQ^a^	6.8	7.3	4.5	4.2	1.9	2.7
**Precision**						
intra-day^b^	–	–	–	1.0	1.2	2.2
inter-day^c^	–	–	–	1.9	1.8	3.0
**Accuracy**^d^						
high spike	98.5	97.8	99.5	102.3	103.3	103.7
medium spike	97.3	97.3	97.3	100.9	101.8	101.8
low spike	97.9	98.5	97.2	98.0	98.8	97.5

a µg/ml.

b Maximum deviation within one day based on peak area in percent.

c Deviation over three days based on peak area in percent.

d Expressed as recovery rate in percent.

**Table 2:** Quantitative results as determined by SFC. Values reflect percent (mg alkaloid/100 mg crude drug), standard deviation are given in parenthesis (n = 3).

Compound	CC-2017-1	CC-2017-2	CC-2017-3	CC-2017-4
quinine (**4**)	1.59 (1.22)	1.89 (0.92)	1.62 (1.34)	1.76 (1.34)
cinchonine (**5**)	2.30 (1.23)	2.16 (0.99)	1.87 (1.55)	2.24 (1.22)
cinchonidine (**6**)	0.90 (0.91)	1.15 (1.47)	1.26 (1.14)	1.05 (0.89)
*Σ*	*4.79*	*5.20*	*4.75*	*5.05*

## References

[1] Achan J, Talisuna AO, Erhart A, Yeka A, Tibenderana JK, Baliraine FN, Rosenthal PJ, D’Alessandro U (2011). Quinine, an old anti-malarial drug in a modern world: role in the treatment of malaria. Malar J.

[2] Sanders NG, Meyers DJ, Sullivan DJ (2014). Antimalarial efficacy of hydroxyethylapoquinine (SN-119) and its derivatives. Antimicrob Agents Chemother.

[3] Horie M, Oishi M, Ishikawa F, Shindo T, Yasui A, Shuzo O, Ito K (2006). Liquid chromatographic analysis of *Cinchona* alkaloids in beverages. J AOAC Int.

[4] Yeboah EMO, Yeboah SO, Singh GS (2011). Recent applications of *Cinchona* alkaloids and their derivatives as catalysts in metal-free asymmetric synthesis. Tetrahedron.

[5] Lajko G, Orosz T, Grecso N, Fekete B, Palko M, Fulop F, Lindner W, Antal P, Ilisz I (2016). High-performance liquid chromatographic enantioseparation of cyclic ß-aminohydroxamic acids on zwitterionic chiral stationary phases based on*Cinchona* alkaloids. Anal Chim Acta.

[6] Gatti R, Gioia MG, Cavrini V (2004). Determination of *Cinchona* alkaloids and vitamin B6 by high-performance liquid chromatography with fluorescence detection. Anal Chim Acta.

[7] Mroczek T, Glowniak K (2000). TLC and HPTLC assay for quinoline and quinuclidine alkaloids in Cinchonae cortex and pharmaceutical preparations. J Planar Chromatogr.

[8] Klein H, Teichmann T (1987). Determination of *Cinchona* alkaloids in pharmaceutical preparations by isotachophoresis (iontophoresis). Pharm Ztg.

[9] Zhao W, Li Y, Zhang Y, Hongfen Y, Yu H, Chen A (2016). Determination of *Cinchona* alkaloids by capillary electrophoresis with novel complex formation. Anal Lett.

[10] Buchberger W, Gstöttenmayr D, Himmelsbach M (2010). Determination of *Cinchona* alkaloids by non-aqueous CE with MS detection. Electrophoresis.

[11] Romon M, Chruszcz-Lipska K, Baranska M (2015). Vibrational analysis of *Cinchona* alkaloids in the solid state and aqueous solutions. J Raman Spectrosc.

[12] Yilmaz A, Nyberg NT, Jaroszewski JW (2012). Extraction of alkaloids for NMR-based profiling: exploratory analysis of an archaic *Cinchona* bark collection. Planta Med.

[13] Fabiano-Tixier AS, Elomri A, Blanckaert A, Seguin E, Petitcolas E, Chemat F (2011). Rapid and green analytical method for the determination of quinoline alkaloids from *Cinchona succirubra* based on microwave-integrated extraction and leaching (MIEL) prior to high performance liquid chromatography. Int J Mol Sci.

[14] McCalley DV (2002). Analysis of *Cinchona* alkaloids by high-performance liquid chromatography and other techniques. J Chromatogr A.

[15] McCalley DV (1990). Quantitative analysis of alkaloids from *Cinchona* bark by high-performance chromatography. Analyst.

[16] Hoffmann CV, Lämmerhofer M, Lindner W (2009). Separation of *Cinchona* alkaloids on a novel strong cation-exchange-type chiral stationary phase – comparison with commercially available strong cation exchanger and reversed-phase packing materials. Anal Bioanal Chem.

[17] Holmfred E, Cornett C, Maldonado C, Ronsted N, Hansen SH (2017). An optimized method for routine separation and quantification of major alkaloids in cortex *Cinchona* by HPLC coupled with UV and fluorescence detection. Phytochem Anal.

[18] Ashraf-Khorassani M, Isaac G, Rainville P, Fountain K, Taylor LT (2015). Study of ultrahigh performance supercritical fluid chromatography to measure free fatty acids without fatty acid ester preparation. J Chromatogr B.

[19] Lesellier E, Latos A, de Oliveira AL (2014). Ultra high efficiency/low pressure supercritical fluid chromatography with superficially porous particles for triglyceride separation. J Chromatogr A.

[20] Giuffrida D, Donato P, Dugo P, Mondello L (2018). Recent analytical techniques advances in the carotenoids and their derivatives determination in various matrixes. J Agric Food Chem.

[21] Huang Y, Zhang T, Zhao Y, Zhou H, Tang G, Fillet M, Crommen J, Jiang Z (2017). Simultaneous analysis of nucleobases, nucleosides and ginsenosides in ginseng extracts using supercritical fluid chromatography coupled with single quadrupole mass spectrometry. J Pharm Biomed Anal.

[22] Yang J, Zhu L, Zhao Y, Xu Y, Sun Q, Liu S, Liu C, Ma B (2017). Separation of furostanol saponins by supercritical fluid chromatography. J Pharm Biomed Anal.

[23] Yang W, Zhang Y, Pan H, Yao C, Hou J, Yao S, Cai L, Feng R (2017). Supercritical fluid chromatography for separation and preparation of tautomeric 7-epimeric spiro oxindole alkaloids from *Uncaria macrophylla*. J Pharm Biomed Anal.

[24] Fu Q, Li Z, Sun C, Xin H, Ke Y, Jin Y, Liang X (2015). Rapid and simultaneous analysis of sesquiterpene pyridine alkaloids from *Tripterygium wilfordii* Hook. f. using supercritical fluid chromatography-diode array detector-tandem mass spectrometry. J Supercrit Fluids.

[25] Hartmann A, Ganzera M (2015). Supercritical fluid chromatography – theoretical background and applications on natural products. Planta Med.

[26] http://www.ich.org/products/guidelines.html.

[27] West C, Lemasson E, Bertin S, Henning P, Lesellier E (2016). An improved classification of stationary phases for ultra-high performance supercritical fluid chromatography. J Chromatogr A.

[28] http://www.waters.com/waters/enUS/SFC-Columns.

[29] Warhurst DC, Craig JC, Adagu IS, Meyer DJ, Lee SY (2003). The relationship of physico-chemical properties and structure to the differential antiplasmodial activity of *Cinchona* alkaloids. Malaria J.

[30] Bamba T (2008). Application of supercritical fluid chromatography to the analysis of hydrophobic metabolites. J Sep Sci.

[31] Grand-Guillaume Perrenoud A, Boccard J, Veuthey JL, Guillarme D (2012). Analysis of basic compounds by supercritical fluid chromatography: attempts to improve peak shape and maintain mass spectrometry compatibility. J Chromatogr A.

[32] Lesellier E, West C (2015). The many faces of packed column supercritical fluid chromatography – a critical review. J Chromatogr A.

[33] Lesellier E (2009). Retention mechanisms in super/subcritical fluid chromatography on packed columns. J Chromatogr A.

[34] Aichner D, Ganzera M (2015). Analysis of anthraquinones in rhubarb (*Rheum palmatum* and *Rheum officinale*) by supercritical fluid chromatography. Talanta.

[35] Murauer A, Ganzera M (2017). Quantitative determination of lactones in *Piper methysticum* (Kava-Kava) by supercritical fluid chromatography. Planta Med.

[36] Desmortreux C, Rothaupt M, West C, Lesellier E (2009). Improved separation of furocoumarins in essential oils by supercritical fluid chromatography. J Chromatogr A.

