# Supplementary material for: Quantitative determination of major alkaloids in Cinchona bark by Supercritical Fluid Chromatography
Source: J Chromatogr A. Author manuscript; Available in PMC 2018 Oct 18. (PMC6193530; doi:10.1016/j.chroma.2018.04.038)
Supplement: Supplementary Information [file NIHMS80100-supplement-Supplementary_Information.docx]

**Optimization of an innovative vinylimidazole-based monolithic stationary phase and its use for pressured capillary electrochromatography**

**SUPPLEMENTARY INFORMATION**

Adele Murauer^1^, Rania Bakry^2^, Gabriel Partl^3^, Christian W. Huck^2^, Markus Ganzera^1^

^1^ Institute of Pharmacy, Pharmacognosy, Center for Molecular Biosciences (CMBI), University of Innsbruck, 6020 Innsbruck, Austria

^2^ Institute for Analytical Chemistry and Radiochemistry, Center for Molecular Biosciences (CMBI), University of Innsbruck, 6020 Innsbruck, Austria

^3^ Institute of General, Inorganic and Theoretical Chemistry, University of Innsbruck, 6020 Innsbruck, Austria

Corresponding Author:

Assoc. Prof. Markus Ganzera, Institute of Pharmacy, Innrain 80-82, 6020 Innsbruck, Austria; e-mail: markus.ganzera@uibk.ac.at; phone: +43-512-507 58406; fax: +43-512-507 58499

**S1)** Structures of monomer and cross linker.

|  | **monomer**  1-vinylimidazole |
| --- | --- |
|  | **cross linker**  3,3’-(hexane-1,6-diyl)bis (1-vinylimidazolium) bromide |

**S2)** IR and NMR-spectra of 3,3’-(hexane-1,6-diyl)bis(1-vinylimidazolium) bromide.

FT-IR


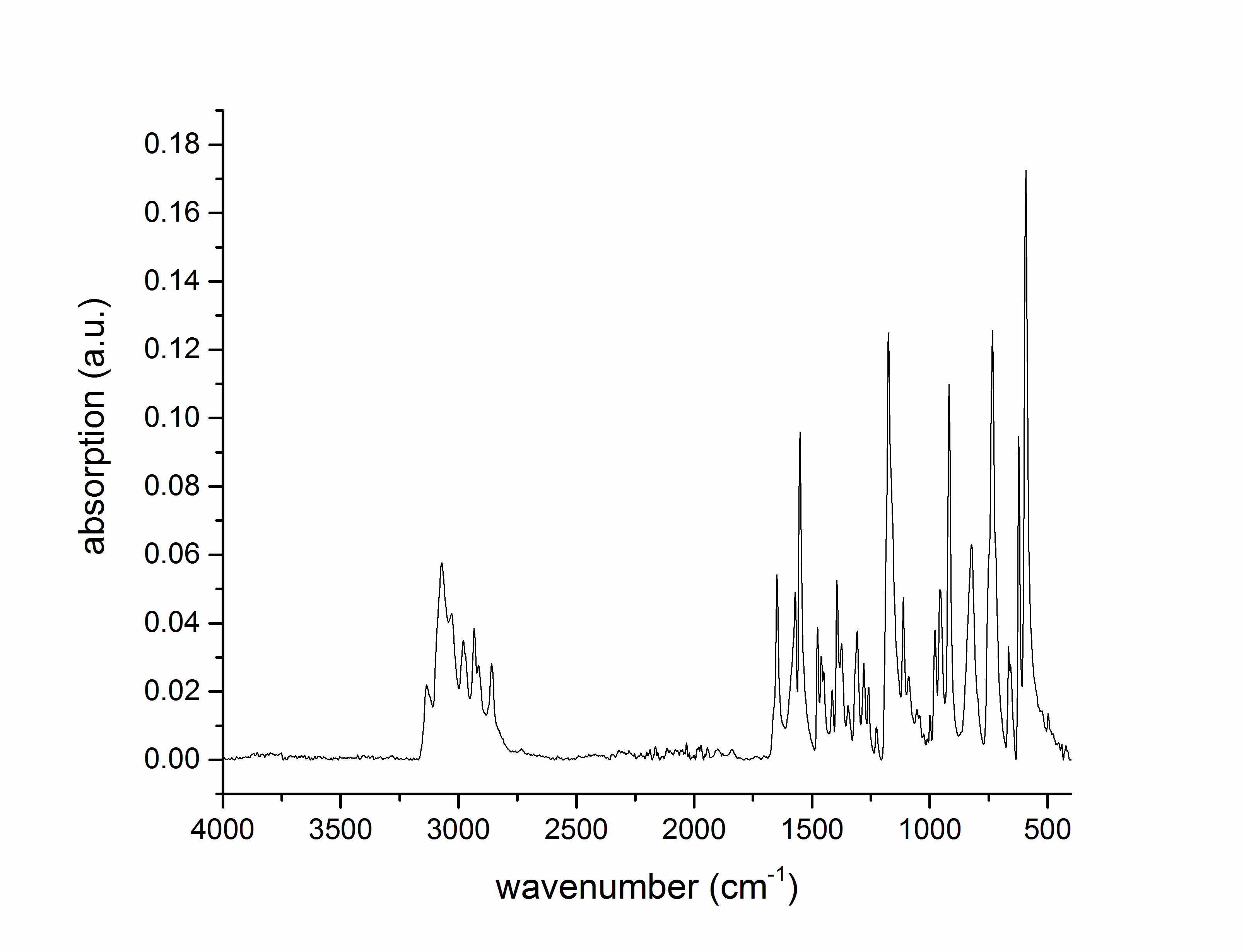


^1^H-NMR (300 MHz, CD_3_OD)


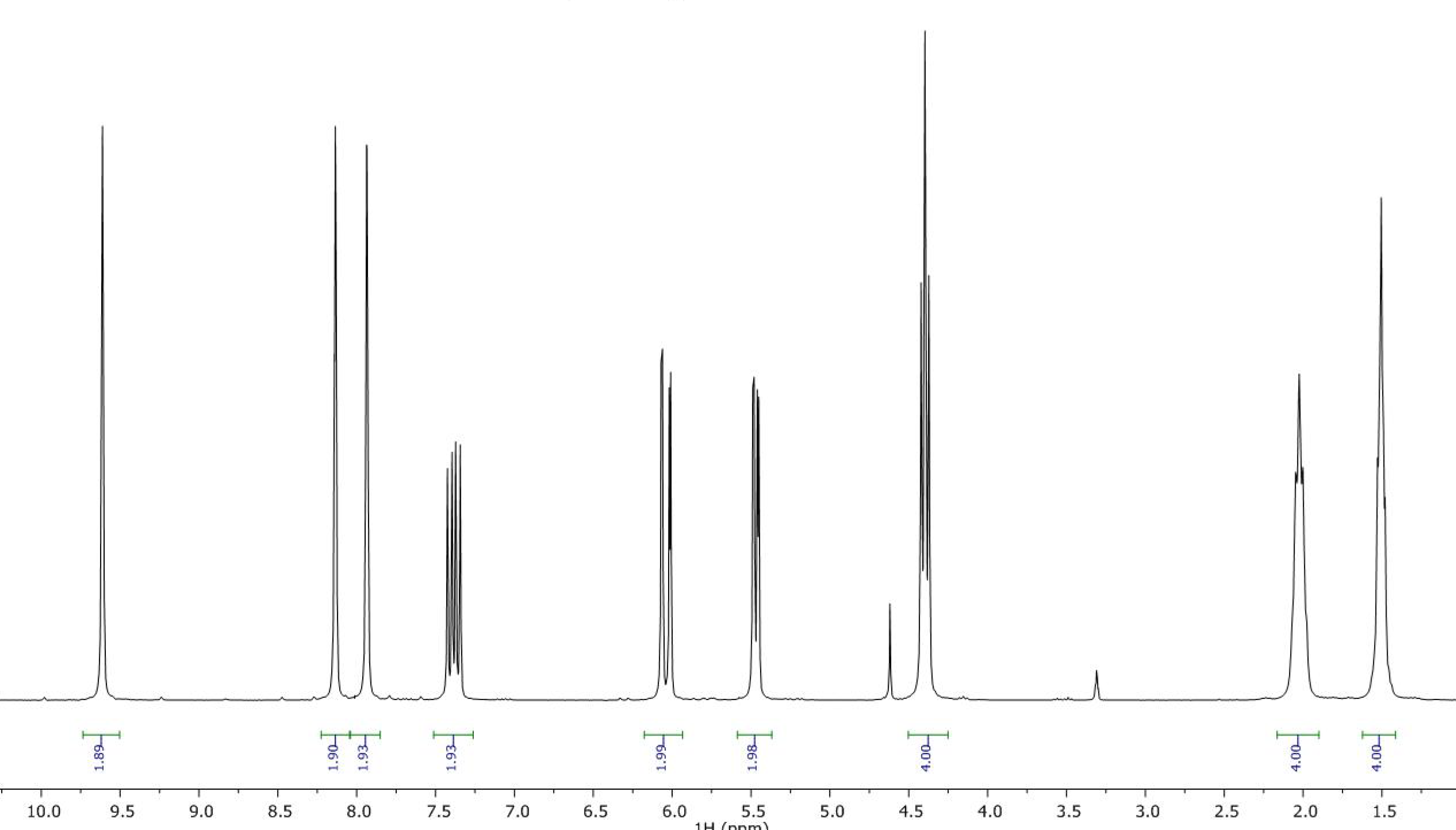


^13^C-NMR (75 MHz, CD_3_OD)


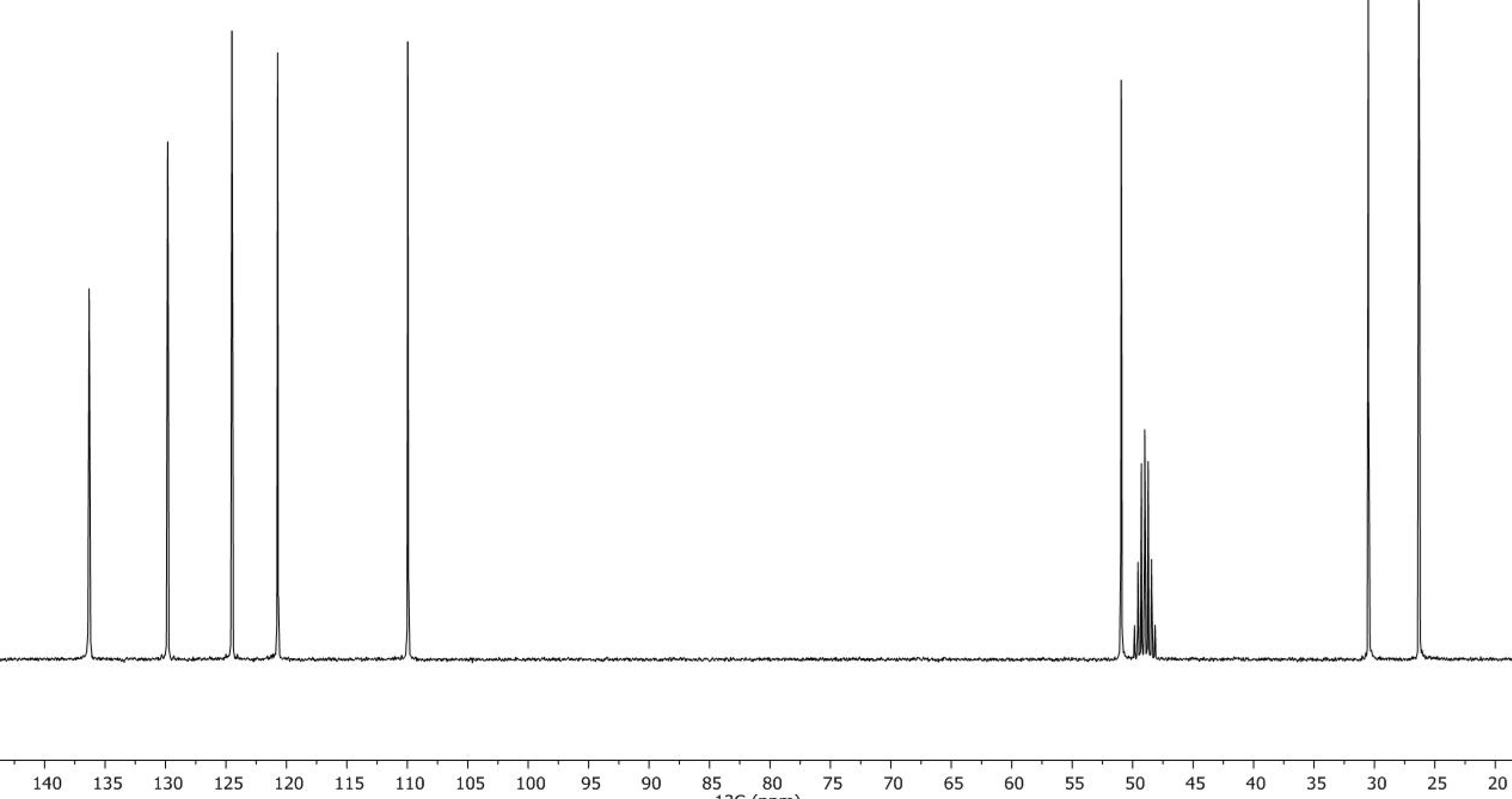


**S3)** Characterization of the finally selected monolithic phase (capillary 4) by FT-IR.


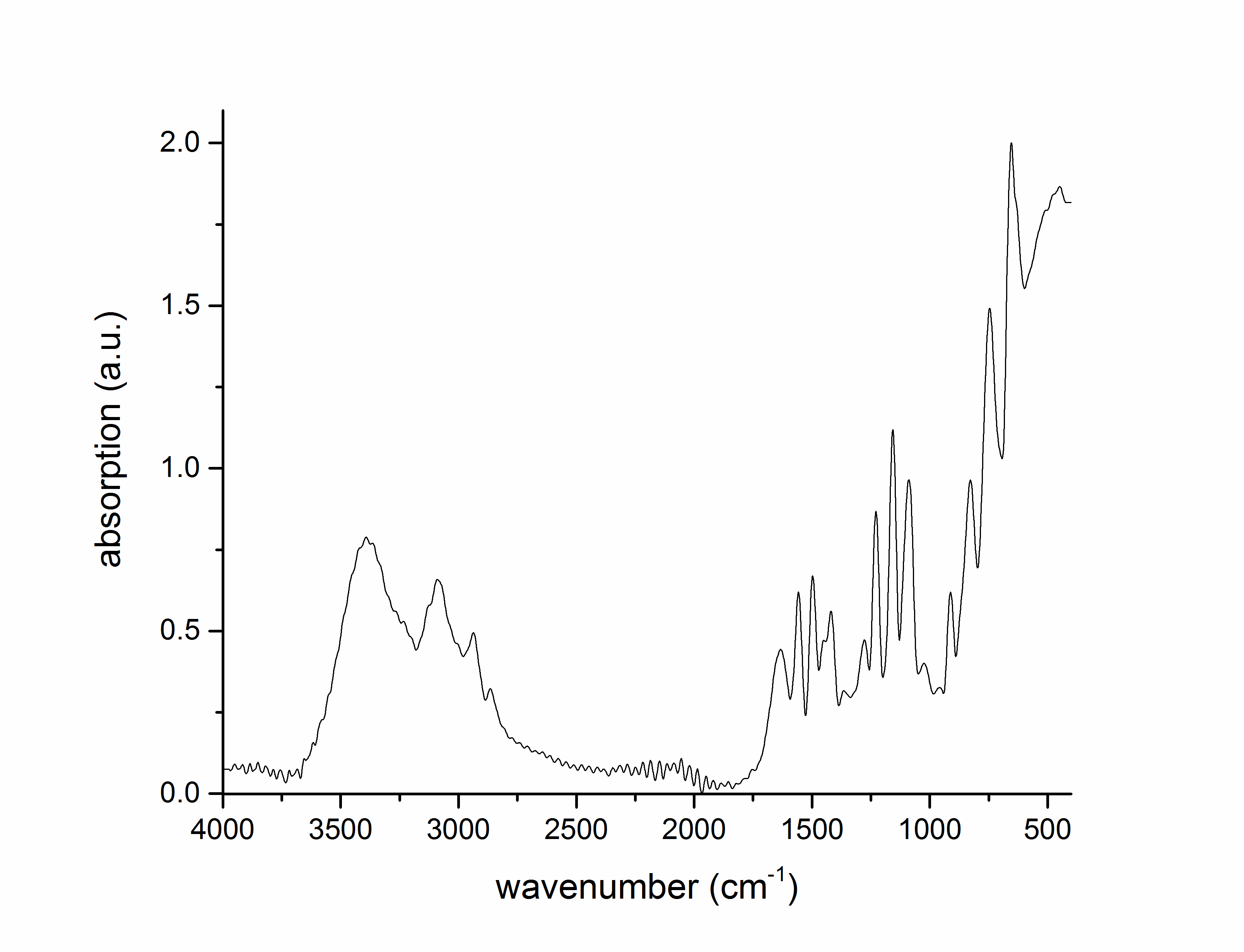


The FT-IR spectrum of the polymeric product is similar to that of the monomer, with the exception of the absence of vinylic bands due to polymerization. Compared to literature, our findings are in good agreement with the data reported for related (polymeric) materials (Lippert et al., Macromolecules, 1985, 18, 63; Feng et al., RSC Adv., 2014, 4, 23389; Katsyuba et al., Helv. Chim. Acta 2004, 87, 2556). The broad bands at 3392 cm^-1^ and 1633 cm^-1^ are likely from water (ν OH, δ OH). Signals at 3090 (ν CH), 1557 (ν CC, ν CN quaternized), 1497 (ν CC, ν CN), 1419 (ν), 1229 (δ CH, ν CN), 1157 (ν CN quaternized), 1089 (δ CH, ν) and 912 (δ, ν) correspond to imidazole (imidazolium) vibrations, bands at 2937 (vas CH, νas CH_2_) and 2865 (νs CH, νs CH_2_) cm^-1^ come from the aliphatic backbone. Peaks at 828, 746 and 654 cm^-1^ are assignable to γ vibrations of both the ring and the backbone.

**S4)** SEM images of capillaries 2, 5, 6 and 7.

| 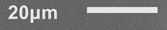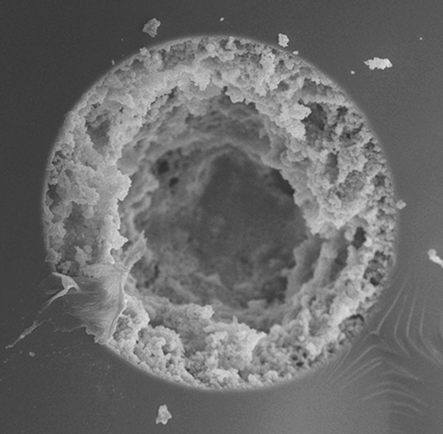 | 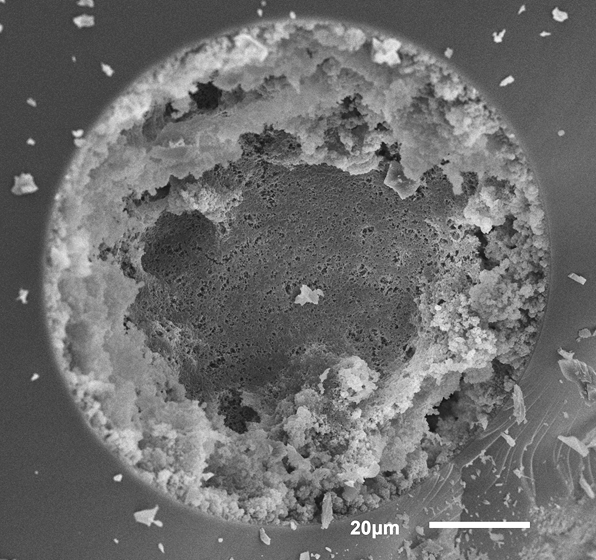 |
| --- | --- |
| capillary 2 | capillary 5 |
| 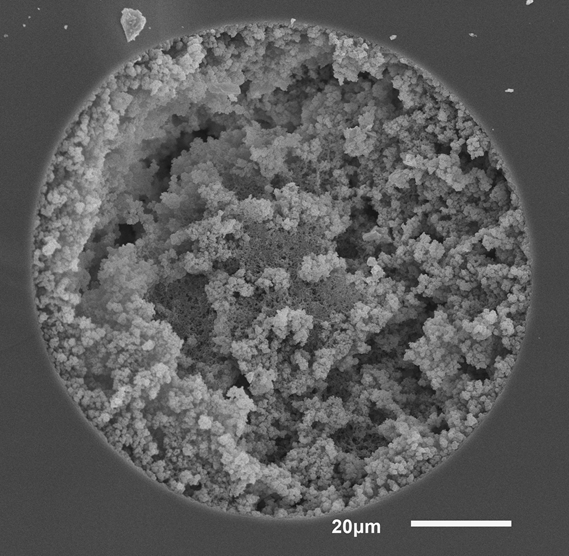 | 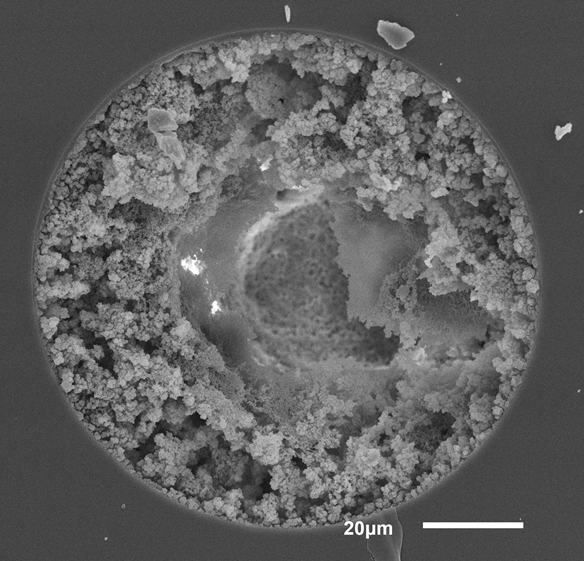 |
| capillary 6 | capillary 7 |

**S5)** Permeability of capillaries 1, 4 and 6 using water, methanol and acetonitrile as mobile phase on a capillary HPLC.

|  |
| --- |
|  |
|  |

**S6)** Structure of standard compounds for CEC method development.

|  |
| --- |
| \|  \| **R_1_** \| **R_2_** \| **R_3_** \| **p*K*a**^a^ \| \| --- \| --- \| --- \| --- \| --- \| \| caffeine (**1**) \| CH_3_ \| CH_3_ \| CH_3_ \| 13.4 \| \| theobromine (**2**) \| H \| CH_3_ \| CH_3_ \| 9.9 \| \| theophylline (**3**) \| CH_3_ \| CH_3_ \| H \| 8.8 \| \| 7-methylxanthine (**4**) \| H \| H \| CH_3_ \| 8.3 \| \| 3-methylxanthine (**5**) \| H \| CH_3_ \| H \| 8.1 \| \| 1-methylxanthine (**6**) \| CH_3_ \| H \| H \| 7.7 \| \| ^a^ according to ChemIDplus database, in aqueous solution \| \| \| \| \| |

**S7)** CE-analysis of EOF marker (acetone, **A**) and standard compounds (**B**) to determine whether analytes are charged at pH 3 or not.


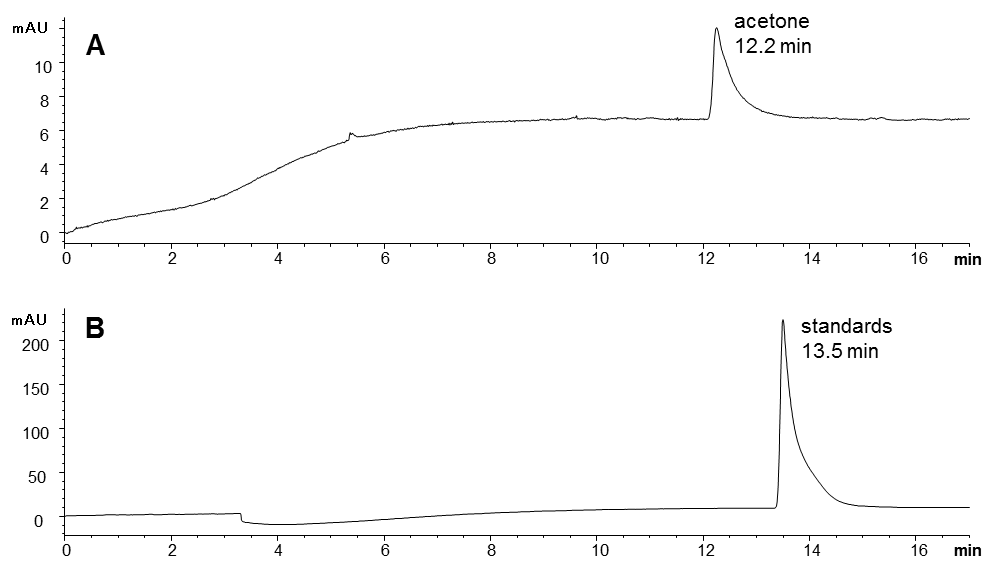


CE conditions: capillary: 50 µm I.D. fused silica with 27.5 cm effective length; buffer: 1:1 mixture of 20 mM citric acid solution with pH 3 and acetonitrile; voltage: + 25 kV; temperature: 30 °C; detection: 280 nm; injection: 50 mbar for 1 sec.
